# Impact of COVID-19 for people living and working with ADHD: A brief review of the literature

**DOI:** 10.3934/publichealth.2021047

**Published:** 2021-08-23

**Authors:** Jack Hollingdale, Nicoletta Adamo, Kevin Tierney

**Affiliations:** 1 Compass, Compass Psychology Services Ltd, London, UK; 2 SCAAND Department, Michael Rutter Centre, South London and Maudsley NHS Trust, London, UK; 3 Department of Child & Adolescent Psychiatry, Institute of Psychiatry, Psychology and Neuroscience, King's College London, London, UK

**Keywords:** ADHD, COVID-19, education provision, service provision, interventions

## Abstract

**Objective:**

COVID-19 lockdowns have changed the social and environmental context. Those with ADHD are more vulnerable to experiencing difficulties than their non-ADHD peers. This paper attempts to provide a brief summary of the literature that has emerged during the COVID-19 pandemic.

**Method:**

A literature search was completed using the following databases; Embase, Ovid Medline, APA PsycInfo. A total of 36 papers were identified as relevant to the topic.

**Results:**

The pandemic has exacerbated the core symptoms of ADHD and co-occurring difficulties. Services have adapted their assessment and intervention protocols for tele-health working and findings suggest that tele-interventions present a viable alternative. However, much of this research utilises small sample sizes and a restricted number of population groups.

**Conclusions:**

More research is required to determine the effectiveness of ADHD care during the pandemic and whether adaptations will be retained post-pandemic.

## 1. Introduction

In 2019, coronavirus 2 (SARS-CoV-2), a severe acute respiratory syndrome, was identified, originating in China [1]. It is known more commonly as COVID-19. By March 2020 the World Health Organisation declared that COVID-19 was a “pandemic”. However, from January 2020 countries began systematic lockdowns, restricting the movement of their citizens. These worldwide restrictions have placed significant stressors on young people and their families, with over three quarters of young people's behaviour and psychology being negatively affected [2]–[4]. At particular risk of the negative effects of COVID-19 social restrictions are those with pre-existing conditions such as Attention Deficit Hyperactivity Disorder (ADHD) [2],[5]–[10].

ADHD is characterised by difficulties with inattention and/or hyperactivity/impulsivity [1],[11]. Additional difficulties are frequently identified to co-occur with ADHD, such as learning difficulties [12], behavioural and emotional difficulties [13],[14], labile mood [15], and sleep difficulties [16],[17].

To determine the impact of COVID-19 lockdown on individuals, their families and professionals, a review of the literature was conducted. At the time of writing this review many parts of the world are still experiencing some degree of social restrictions and therefore the experiences of lockdown will be referred to in the present tense.

## 2. Methods

**Figure 1.**
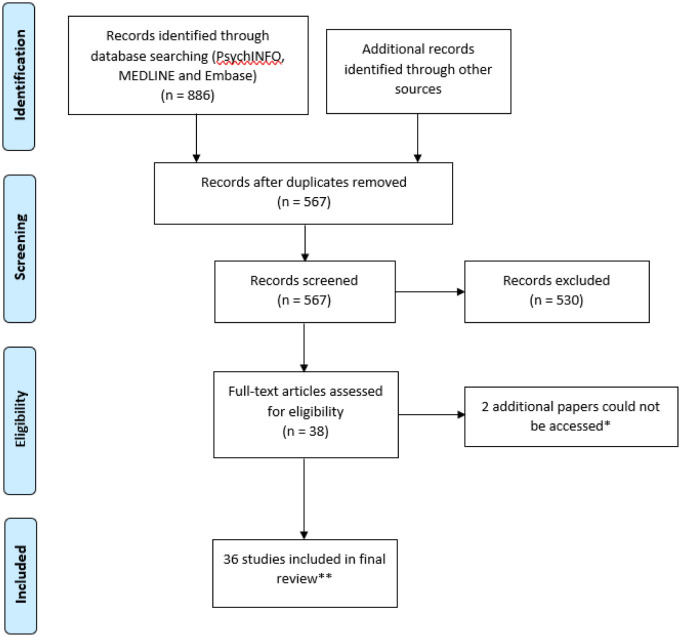
Search strategy [18]. Note: * Sederstrom J (2020) Pandemic presents unique challenges: Caregivers of children with ADHD can choose from an increasing number of treatments that can be customized. Drug Topics, 164: 42–43. And Valli A, Mauri V, Vanzin L, et al. (2020) The challenge of telepsychiatry in childhood: The parent training for ADHD. Giornale di Neuropsichiatria dell'Eta Evolutiva, 40: 148–153. **See Table 1 for list of included papers and Table 2 for summary of paper outcomes.

A literature search in Embase, Ovid Medline, APA PsycInfo was completed on 18^th^ March 2021. No restrictions on country of origin or language were enforced. All articles that reported information about young people and/or adults with a diagnosis of ADHD or ADHD symptoms were included. In addition, articles that explored the effect of COVID-19 on parents or caregivers of individuals with ADHD were included. This review was also interested in exploring adaptations to neurodevelopmental or mental health services and educational providers. The following terms were used to identify papers that included ADHD: “ADHD” or “Attention Deficit Hyperactivity Disorder” or “Hyperactivity Disorder” or “ADD” or “Attention Deficit Disorder” or “Hyperkinetic Disorder”. This resulted in 364,658 results. Published papers relating to COVID-19 were identified using the following search terms: “COVID-19” and “coronavirus”. This resulted in 262,069 results. When these search strings were combined, 886 papers were identified. 319 duplicates were removed leaving 567 papers. The titles and abstracts of these 567 papers were screened and a total of 36 papers were included. Papers were excluded if they did not explore the effect of COVID-19 on ADHD symptomology, the functioning of people with ADHD or service and/or other institutional provisions for people with ADHD. See Figure 1 for search strategy and Tables 1–2 for list of included papers. The bibliographies of these papers were reviewed to identify further relevant papers, but none were identified.

**Table 1.** Summary of studies included in the literature review.

No.	Study	Country	Clinical or Community	ADHD Sample Size	Age of Sample
1.	Adamou M, Fullen T, Galab N, et al. (2020) Psychological effects of the covid-19 imposed lockdown on adults with attention deficit/hyperactivity disorder: cross-sectional survey study. *JMIR Formative Research* 4: e24430.	UK	Clinical	24	Mean = 21.75
2.	Ando M, Takeda T, Kumagai K (2021) A qualitative study of impacts of the COVID-19 pandemic on lives in adults with attention deficit hyperactive disorder in Japan. *International Journal of Environmental Research and Public Health* 18: 1–10.	Japan	Clinical	4	20–40s
3.	Arbel Y, Fialkoff C, Kerner A, et al. (2020) Can Increased Recovery Rates from Coronavirus be explained by Prevalence of ADHD? An Analysis at the US Statewide Level. *Journal of attention disorders* 1087054720959707.	Israel/US	Community	NR	NR
4.	Becker SP, Breaux R, Cusick CN, et al. (2020) Remote Learning During COVID-19: Examining School Practices, Service Continuation, and Difficulties for Adolescents With and Without Attention-Deficit/Hyperactivity Disorder. *Journal of Adolescent Health* 67: 769–777.	US	Community	238	15.64–17.99
5.	Becker SP, Dvorsky MR, Breaux R, et al. (2021) Prospective examination of adolescent sleep patterns and behaviors before and during covid-19. *Sleep*.	US	Community	58	Mean = 16.27
6.	Becker SP, Gregory AM (2020) Editorial Perspective: Perils and promise for child and adolescent sleep and associated psychopathology during the COVID-19 pandemic. *Journal of child psychology and psychiatry, and allied disciplines* 61: 757–759.	US	NA	NA	NA
7.	Bobo E, Lin L, Acquaviva E, et al. (2020) How do children and adolescents with Attention Deficit Hyperactivity Disorder (ADHD) experience lockdown during the COVID-19 outbreak? *Encephale* 46: S85–S92.	France	Community	533 (Parents)	Mean = 10.5
8.	Breaux R, Dvorsky MR, Marsh NP, et al. (2021) Prospective impact of COVID-19 on mental health functioning in adolescents with and without ADHD: protective role of emotion regulation abilities. *Journal of child psychology and psychiatry, and allied disciplines, no pagination*.	US	NR	118	15–17
9.	Çetin FH, Ucar HN, Türkoğlu S, et al. (2020) Chronotypes and trauma reactions in children with ADHD in home confinement of COVID-19: full mediation effect of sleep problems. *Chronobiology international* 37: 1214–1222.	Turkey	Clinical	76	8–12
10.	Cortese S, Asherson P, Sonuga-Barke E, et al. (2020) ADHD management during the COVID-19 pandemic: guidance from the European ADHD Guidelines Group. *The Lancet Child and Adolescent Health* 4: 412–414.	NA	NA	NA	NA
11.	Cortese S, Coghill D, Santosh P, et al. (2020) Starting ADHD medications during the COVID-19 pandemic: recommendations from the European ADHD Guidelines Group. *The Lancet Child and Adolescent Health* 4: e15.	NA	NA	NA	NA
12.	DiBacco TA, Gaynor ST (2021) Prolonged Exposure Therapy: A Case of Comorbid PTSD, ADHD, and GAD. *Clinical Case Studies*.	US	Clinical	Case Study	29
13.	Fogler JM, Normand S, O'Dea N, et al. (2020) Implementing Group Parent Training in Telepsychology: Lessons Learned During the COVID-19 Pandemic. *Journal of pediatric psychology* 45: 983–989.	US	Clinical	20	5–11
14.	Kavoor AR, Mitra S (2021) Managing Attention Deficit Hyperactivity Disorder during COVID-19 Pandemic. *Journal of Neurosciences in Rural Practice* 12: 1–2.	NA	NA	NA	NA
15.	Laslo-Roth R, Bareket-Bojmel L, Margalit M (2020) Loneliness experience during distance learning among college students with ADHD: the mediating role of perceived support and hope. *European Journal of Special Needs Education*.	Israel	Community	119	Mean = 26.79
16.	Low DM, Rumker L, Talkar T, et al. (2020) Natural language processing reveals vulnerable mental health support groups and heightened health anxiety on reddit during COVID-19: Observational study. *Journal of Medical Internet Research* 22.	World	Community	NR	18–29
17.	Mallik CI, Radwan RB (2021) Impact of lockdown due to covid-19 pandemic in changes of prevalence of predictive psychiatric disorders among children and adolescents in bangladesh. *Asian Journal of Psychiatry* 56: 102554.	Bangladesh	Community	NR	4–17
18.	McGowan G, Conrad R. Potts H (2020) 51.2 Challenges With Managing Children And Adolescents With Adhd During The Covid-19 Pandemic: A Review Of The Literature. *Journal of the American Academy of Child and Adolescent Psychiatry* 59: S251.	NA	NA	NA	NA
19.	McGrath J (2020) ADHD and Covid-19: Current roadblocks and future opportunities. *Irish Journal of Psychological Medicine* 37: 204–211.	NA	NA	NA	NA
20.	Melegari MG, Giallonardo M, Sacco R, et al. (2021) Identifying the impact of the confinement of Covid-19 on emotional-mood and behavioural dimensions in children and adolescents with attention deficit hyperactivity disorder (ADHD). *Psychiatry Research* 296.	Italy	Community	992	Mean = 11.52
21.	Nissen JB, Hojgaard DRMA, Thomsen PH (2020) The immediate effect of COVID-19 pandemic on children and adolescents with obsessive compulsive disorder. *BMC Psychiatry* 20.	Denmark	Clinical and Community	19	Mean = 14.9
22.	Nonweiler J, Rattray F, Baulcomb J, et al. (2020) Prevalence and associated factors of emotional and behavioural difficulties during covid-19 pandemic in children with neurodevelopmental disorders. *Children* 7.	UK	Community	183	4–15
23.	Oddo LE, Garner A, Novick DR, et al. (2021) Remote Delivery of Psychosocial Intervention for College Students with ADHD during COVID-19: Clinical Strategies, Practice Recommendations, and Future Considerations. *Evidence-Based Practice in Child and Adolescent Mental Health* 6: 99–115.	USA	Community	10	NR
24.	Palacio-Ortiz JD, Londono-Herrera JP, Nanclares-Marquez A, et al. (2020) Psychiatric disorders in children and adolescents during the COVID-19 pandemic. *Revista Colombiana de Psiquiatria* 49: 279–288.	NA	NA	NA	NA
25.	Panda PK, Gupta J, Chowdhury SR, et al. (2020) Psychological and Behavioral Impact of Lockdown and Quarantine Measures for COVID-19 Pandemic on Children, Adolescents and Caregivers: A Systematic Review and Meta-Analysis. *Journal of Tropical Pediatrics*.	NA	NA	NA	NA
26.	Salinas CM, Bordes Edgar V, Berrios Siervo G, et al. (2020) Transforming pediatric neuropsychology through video-based teleneuropsychology: an innovative private practice model pre-COVID-19. *Archives of clinical neuropsychology : the official journal of the National Academy of Neuropsychologists* 35: 1189–1195.	US	Clinical	NA	Mean = 10.2
27.	Saline S (2021) Thriving in the New Normal: How COVID-19 has Affected Alternative Learners and Their Families and Implementing Effective, Creative Therapeutic Interventions. *Smith College Studies in Social Work*, 1–28.	NA	NA	NA	NA
28.	Sciberras E, Patel P, Stokes MA, et al. (2020) Physical Health, Media Use, and Mental Health in Children and Adolescents With ADHD During the COVID-19 Pandemic in Australia. *Journal of Attention Disorders*, 1087054720978549.	Australia	Community	213 (Parents)	5–17
29.	Shah AC, Badawy SM (2021) Telemedicine in pediatrics: systematic review of randomized controlled trials. *JMIR Pediatrics and Parenting* 4: e22696.	NA	NA	NA	NA
30.	Shah R, Raju VV, Sharma A, et al. (2021) Impact of COVID-19 and Lockdown on Children with ADHD and Their Families-An Online Survey and a Continuity Care Model. *Journal of Neurosciences in Rural Practice* 12: 71–79.	NA	NA	NA	NA
31.	Sibley MH, Ortiz M, Gaias LM, et al. (2021) Top problems of adolescents and young adults with ADHD during the COVID-19 pandemic. *Journal of Psychiatric Research* 136: 190–197.	US	Community	134	Mean = 17.11
32.	Summers J, Baribeau D, Mockford M, et al. (2021) Supporting Children With Neurodevelopmental Disorders During the COVID-19 Pandemic. *Journal of the American Academy of Child and Adolescent Psychiatry* 60: 2–6.	NA	NA	NA	NA
33.	Valentine AZ, Hall SS, Young E, et al. (2021) Implementation of telehealth services to assess, monitor, and treat neurodevelopmental disorders: Systematic review. *Journal of Medical Internet Research*, 23.	NA	NA	NA	NA
34.	Wallis KE, Mule C, Mittal S, et al. (2020) Use of Telehealth in Fellowship-Affiliated Developmental Behavioral Pediatric Practices During the COVID-19 Pandemic. *Journal of developmental and behavioral pediatrics: JDBP*.	US	Clinical and Community	NA	NA
35.	Wang Q, Xu R, Volkow ND (2021) Increased risk of COVID-19 infection and mortality in people with mental disorders: analysis from electronic health records in the United States. *World Psychiatry* 20: 124–130.	US	Community	400	18–65
36.	Zhang J, Shuai L, Yu H, et al. (2020) Acute stress, behavioural symptoms and mood states among school-age children with attention-deficit/hyperactive disorder during the COVID-19 outbreak. *Asian Journal of Psychiatry*, 51.	China	Community	241 (Parents)	Mean = 9.43

Note: NR = Not Reported; NA = Not applicable.

**Table 2.** ADHD specific outcome summary of studies included in the literature review.

No.	Study	Study Outcomes
1.	Adamou M, Fullen T, Galab N, et al. (2020).	The adults with ADHD surveyed had significant levels of emotional distress during the COVID-19 pandemic period. However, there was no evidence of significant deterioration to the mental health of the sample during the COVID-19 pandemic.
2.	Ando M, Takeda T, Kumagai K (2021).	The COVID-19 pandemic could be a factor in inducing psychological distress in the participants who adjust relatively better at work/school but did not do well at home before the pandemic. The study indicates the need for special support for individuals with ADHD, especially those who originally had difficulties at home.
3.	Arbel Y, Fialkoff C, Kerner A, et al. (2020).	Based on information from 2011 regarding the prevalence of *ADHD* across the US by state, findings suggest that, while there are no correlations between *ADHD* and population size, infection and mortality rates from coronavirus, recovery rates (recovery-population ratio) *rise* with the prevalence of *ADHD. ADHD* might provide an evolutionary advantage.
4.	Becker SP, Breaux R, Cusick CN, et al. (2020).	This study provides initial findings of the nature and impact of remote learning during the COVID-19 pandemic. Adolescents, particularly those with mental health and/or learning difficulties require additional support from schools and communities.
5.	Becker SP, Dvorsky MR, Breaux R, et al. (2021).	COVID-19 had negative and positive impacts on adolescent sleep. Adolescents with ADHD did not experience the benefit of increased school night sleep duration during COVID-19 like adolescents without ADHD. Negative affect and health behaviours may be useful intervention targets for reducing negative impacts of COVID-19 for adolescent sleep.
6.	Becker SP, Gregory AM (2020).	Provides a summary of the positive and negative impact on sleep during COVID-19. It also provides considerations for research and practice.
7.	Bobo E, Lin L, Acquaviva E, et al. (2020).	According to parents, most children and adolescents with ADHD experience stability or improvement of their well-being. An improvement in school-related anxiety and the flexible adjustment to the children's rhythms as well as parents' increased awareness of the difficulties their children experience were among the key topics in parents' descriptions.
8.	Breaux R, Dvorsky MR, Marsh NP, et al. (2021).	The early observed increases in adolescent mental health symptoms during the COVID-19 pandemic do not on average appear to be sustained following the lift of stay-at-home orders. Emotion dysregulation and ADHD increase the risk for sustained negative mental health functioning and highlight the need for interventions for these populations during chronic stressors.
9.	Çetin FH, Ucar HN, Türkoğlu S, et al. (2020).	Findings indicate that chronotype plays an important role on the negative effects of home confinement of ADHD children during the COVID-19 outbreak.
10.	Cortese S, Asherson P, Sonuga-Barke E, et al. (2020).	Findings indicate that strategies routinely recommended in parent focused ADHD interventions, as well as mental-wellbeing interventions for children and young people are completed. The risks and benefits of initiating or maintaining medication under the COVID-19 restrictions implemented in some countries should be carefully considered.
11.	Cortese S, Coghill D, Santosh P, et al. (2020).	Presents the European ADHD Guidelines Group (EAGG) perspective on starting ADHD medications (specifically psychostimulants and atomoxetine), during the pandemic, for patients who did not have a baseline, face-to-face cardiovascular assessment before the crisis began.
12.	DiBacco TA, Gaynor ST (2021).	This case study displays the successful application of Prolonged Exposure (PE) for a client with diagnoses of Posttraumatic Stress Disorder (PTSD), ADHD, and Generalized Anxiety Disorder (GAD). The case study illustrates a positive synergy between psychostimulant treatment and PE.
13.	Fogler JM, Normand S, O'Dea N, et al. (2020).	Telepsychology “Bootcamp” for ADHD can be implemented with comparably high levels of content and process fidelity and treatment satisfaction to in-person groups; and it appears to be feasible and acceptable to caregivers. Caregiver and clinician qualitative feedback revealed themes of appreciating the convenience of telepsychology, while experiencing some challenges in relating to others and sharing over video.
14.	Kavoor AR, Mitra S (2021).	Provides a brief summary of the current literature on the management of ADHD during COVID-19 and signposts areas for more research.
15.	Laslo-Roth R, Bareket-Bojmel L, Margalit M (2020).	Students with ADHD reported higher levels of loneliness and more negative experiences with distance learning than their peers. Results demonstrated that ADHD and negative experiences with distance learning predicted higher levels of loneliness, while college support and peer support in addition to hopeful thinking mediated these relations.
16.	Low DM, Rumker L, Talkar T, et al. (2020).	The ADHD group had one of the highest increases in negative semantic features for certain subreddits. Some parents in France of children and adolescents diagnosed with ADHD reported increased hyperactivity and inattention, while other parents reported symptomatic improvement.
17.	Mallik CI, Radwan RB (2021).	Prevalence of emotional, conduct disorder and hyperactivity were also increased significantly during the lockdown period than before. Conduct disorder and hyperactivity were more prevalent among boys both before and within lockdown.
18.	McGowan G, Conrad R, Potts H (2020).	The risk for worsening ADHD symptoms under quarantine highlighted the need for more home-based interventions and symptom monitoring by families and providers. Further research is needed to determine the efficacy of telehealth services during the pandemic.
19.	McGrath J (2020).	There is a growing evidence base for telepsychiatry in assessing and treating young people with ADHD. This paper provides a practical approach that could be considered by CAMHS nationally.
20.	Melegari MG, Giallonardo M, Sacco R, et al. (2021).	ADHD Subjects with previous low severity degree of conduct and emotional problems significantly worsened in almost all dimensions during the lockdown. On the contrary, ADHD patients with moderate and severe degree showed important improvement during the lockdown.
21.	Nissen JB, Hojgaard DRMA, Thomsen PH (2020).	Participants with OCD experienced a worsening of their OCD, anxiety, and depressive symptoms during the pandemic. The worsening was most pronounced in children with early age of onset and a family history of attention deficit hyperactivity disorder.
22.	Nonweiler J, Rattray F, Baulcomb J, et al. (2020).	Young people with neurodevelopmental conditions, compared to neurotypical controls, had a higher prevalence of emotional symptoms and conduct problems and fewer prosocial behaviours. All groups had worse emotional symptoms than pre-COVID groups, and those with attention-deficit/hyperactivity disorder showed inflated conduct problems.
23.	Oddo LE, Garner A, Novick DR, et al. (2021).	Using advancements in technology, the SUCCEEDS program provides individual and group psychosocial services to college students with ADHD via telehealth. The SUCCEEDS program generated novel and creative clinical strategies to assist students in problem solving, adaptive coping, organizational skills, and time management strategies
24.	Palacio-Ortiz JD, Londono-Herrera JP, Nanclares-Marquez A, et al. (2020).	This paper provides a summary of the literature on the effect of the pandemic on children and adolescents with previous psychiatric disorders including ADHD.
25.	Panda PK, Gupta J, Chowdhury SR, et al. (2020).	A systematic review and meta-analysis revealed that children with pre-existing behavioural problems like autism and ADHD have a high probability for the worsening of their behavioural symptoms
26.	Salinas CM, Bordes Edgar V, Berrios Siervo G, et al. (2020).	Video based teleneuropsychology benefits consumers through reduced wait times, decreased family financial burden (i.e. travel and parent time off work), expedites referrals for interventions and increases access for those whose access is limited by geography, language and culture.
27.	Saline S (2021).	Interventions to help families of young people who are neurodiverse are most effective when they rely on the 5 C's method of successful ADHD parenting. Working together for effective solutions based on meaningful incentives reduces family conflict, improves young people's participation and fosters parent-child cooperation.
28.	Sciberras E, Patel P, Stokes MA, et al. (2020).	Compared to pre-pandemic, children with ADHD had less exercise, less outdoor time, and less enjoyment in activities, while television, social media, gaming, sad/depressed mood, and loneliness were increased. Child stress about COVID-19 restrictions was associated with poorer functioning across most domains. Most parents reported positive changes for their child including more family time.
29.	Shah AC, Badawy SM (2021).	The evidence from this review suggests that telemedicine services for the general public and paediatric care are comparable to or better than in-person services.
30.	Shah R, Raju VV, Sharma A, et al. (2021).	During the lockdown period, there was worsening of symptoms of ADHD in the form of increase (slight or marked) in the activity level, irritability, and disturbing or disruptive behaviour in children. In terms of behaviour of family members, there was marked/slight increase in irritability, and shouting at the child, verbal abuse, and punishing the child. Additionally, there was an increase in the praising and spending time with the child.
31.	Sibley MH, Ortiz M, Gaias LM, et al. (2021).	For adolescents and young people with ADHD, several risk factors for depression and school dropout were incurred during the early months of the COVID-19 pandemic. adolescents and young people with ADHD should be monitored for school disengagement and depressive symptoms during the COVID-19 pandemic
32.	Summers J, Baribeau D, Mockford M, et al. (2021).	This service evaluation indicated that the program provided by a neurodevelopmental clinic was well received, the virtual format and technology ran smoothly, and the recommendations were generally perceived as helpful.
33.	Valentine AZ, Hall SS, Young E, et al. (2021).	This systematic review identified that telehealth has the potential to increase treatment availability, decrease diagnosis waiting times, and aid in the monitoring of neurodevelopmental conditions.
34.	Wallis KE, Mule C, Mittal S, et al. (2020).	This study identified that most sites are providing evaluations and ongoing care for developmental behavioural paediatric conditions through telehealth.
35.	Wang Q, Xu R, Volkow ND (2021).	Women with a recent diagnosis of a mental disorder had higher odds of COVID-19 infection than men after adjusting for age, ethnicity and medical comorbidities, with the strongest gender disparity for ADHD. Age had significant effects on COVID-19 risk, after adjusting for gender, ethnicity and medical comorbidities, among patients with a recent diagnosis of ADHD.
36.	Zhang J, Shuai L, Yu H, et al. (2020).	Children's ADHD behaviours were significantly worsened during the pandemic in comparison to their normal state. Children's overall mood, parents' overall mood state, and children's study time, significantly predicted children's ADHD behaviours.

## 3. Results

### 3.1. ADHD symptoms, co-occurring difficulties and impairment

Adults with ADHD are identified to have experienced significant levels of emotional and behavioural difficulties during the pandemic but the degree of deterioration is uncertain [19],[20]. However, the pandemic is reported to have exacerbated difficulties for many young people with ADHD and their families [5] and rates of ADHD have been reported to have increased during the pandemic compared with pre-pandemic rates [21]. Shah and colleagues [22] found that during lockdown young people with ADHD experienced an increase in activity, disruptive behaviour and irritability. Symptoms of inattention and oppositionality have also been found to increase for young people with ADHD compared with their non-ADHD counterparts [23].

Regarding mental health, low mood and isolation have been found to increase for young people with ADHD as a result of COVID-19 restrictions [24]. In addition, a decrease in general well-being, demonstrated by an increase in oppositionality and emotional outbursts have been reported by parents [7],[25]. The presence of both ADHD and emotional dysregulation are found to increase the risk of negative mental health functioning [23].

Young people with Obsessive Compulsive Disorder (OCD) and a family history of ADHD are also reported to have experienced an increase of OCD symptoms during lockdown [26]. Furthermore, young people with ADHD, experience a reduction in outdoor activities and an increase in indoor activities, such as social media use, watching television and video game play [24] which can be detrimental to well-being and functioning for some young people [27].

Young people are reported to be experiencing sleep difficulties during lockdown due to symptoms of COVID-19, sedentary behaviours, isolation, limited exposure to sunlight, poor sleep hygiene and increased exposure to “blue light” [28]–[30]. However, young people with ADHD may be at a higher risk of sleep difficulties due to medication use and co-occurring conditions [31] which may in turn increase or mediate other difficulties such as inattention, emotional difficulties and conduct problems [32],[33].

### 3.2. Service provision

National lockdowns and restrictions on face-to-face contact have had a dramatic impact on the services that are provided by institutions and the mode by which these services are provided. Given the high transmission rates of COVID-19 [34] and the fact that the majority of young people are asymptomatic [35], face-to-face contact was significantly reduced and, in many cases, stopped altogether and a number of adaptations were developed for both young people and adult services.

### 3.3. Assessments

Referral rates for ADHD assessments have reduced by as much as 80% in Ireland [36] and this has been attributed to the reduction in school referrals and families minimising non-emergency contact with health care services.

Tele-assessments have been identified as effective in the diagnosing and management of neurodevelopmental disorders, including ADHD [37],[38], but less is known about the accuracy of these assessments for adults compared with face-to-face assessments [37].

Diagnostic assessments that require liaison with schools has been impaired or delayed by the closure of many schools and the capacity of educators. Whilst screening measures can be facilitated remotely either by post or email it has not been possible to undertake behavioural observations when required or monitor the effect of medication within the school environment [36].

### 3.4. Interventions

#### 3.4.1. Pharmacological interventions

The National Institute for Health and Care Excellence [39] recommends a baseline physical health examination prior to starting ADHD medications which has not been possible as many services are limited to remote appointments.

Due to concerns over the potential increase in health risks for individuals with ADHD if medication is not initiated or they fail to access their existing prescriptions, the European ADHD Guidelines Group (EAGG) recommended that it is appropriate to start ADHD medication remotely, with specific stipulations [40].

As a result of lockdown restrictions, additional responsibility has been placed on families to ensure that their dependants attend clinical examinations when required, are compliant with their medication and engage in appropriate home-based monitoring. For some families this may increase financial pressures, such as having to purchase blood pressure machines.

#### 3.4.2. Psycho-social interventions

Given the restrictions around face-to-face interventions, adapted remote psycho-social interventions present a promising alternative to the traditional models for a range of conditions [41]–[47], and this is also true for ADHD [48]. Examples of successful ADHD psychosocial interventions that have been delivered remotely include a parent training group [49], a programme for college students [50] and a brief intervention programme [51]. Overall, tele-interventions have been identified to have a number of possible benefits including, reduced wait times, providing access to more geographically remote populations, and reduced financial stressors, such as travel or taking time off work [52]. However, it should be noted that these studies include relatively small sample sizes and far more research is required to determine the effectiveness of remote tele-interventions across the full range of therapies and programmes.

As a result of the restricted access to face-to-face interventions for young people with ADHD, parents are taking on more of the responsibility for psycho-social interventions. For example, using behavioural parenting strategies and self-help versions of evidenced based interventions [53].

#### 3.4.3. Educational provision

The transition to remote study has posed challenges for learning institutions worldwide [54]. However, for students with ADHD, this medium of learning is particularly problematic [9],[55]–[58]. For example, Laslo-Roth and colleagues [59] found that students with ADHD reported higher levels of loneliness and more negative experiences with distance learning than non-ADHD peers, including fewer routines [60]. However, peer and college support along with hopeful thinking mediated feelings of loneliness and negative experiences [59]. Families of young people with ADHD are also reported to have experienced difficulties managing remote learning due to a reduction in school support and additional financial costs, such as improving their internet plans [60]. The hereditary nature of ADHD may mean that parents and carers who are taking responsibility for their children's learning may also be struggling with their own experiences of inattention, hyperactivity and impulsivity. In some cases, children have continued to attend school due to individual or family necessity. It is not known to what extent smaller class sizes may have positively or negatively affected children with ADHD.

#### 3.4.4. Positives

There is emerging evidence that lockdown has not had a negative impact and in some cases, it has actually been helpful for young people with ADHD [25],[61]. Bobo and colleagues [25] found that approximately a third of young people were not reported to have experienced any change in their well-being since lockdown and around a third reported improvements in their well-being. Parents attributed this to less stress associated with school attendance and structure. In addition, parents thought that their children were subject to less criticism or punishment at school which had fostered more positive views about the self. Parents of young people with ADHD have also reported an increase in family time as a positive impact of lockdown [24].

ADHD has been found to be a risk factor for contracting COVID-19 [62], particularly for females [63], partially attributed to the non-adherence of government instructions such as social distancing or wearing masks [64]. However, in a US study, recovery rates (recovery-population ratio) have been found to rise with the prevalence of ADHD [65] suggesting that ADHD may present as a possible protective factor against severe COVID-19 symptoms.

## 4. Discussion

Individuals, families and services have had to respond quickly and adapt to familial, social and educational changes. Individuals with ADHD are more likely to be at risk of contracting COVID-19 and are more likely to experience difficulties due to lockdown with both the core features of ADHD and co-occurring mental health difficulties. These difficulties also appear to be more severe than their non-ADHD counterparts. However, it should be noted that some benefits have been observed for young people with ADHD.

Services have made attempts to adapt their assessment and intervention provisions with partial success. As might be expected, referrals for assessments and the completion of assessments have reduced during the pandemic. As a result, it is likely that services will need to be prepared for a spike in ADHD referrals following the end of the pandemic and extended waitlists will need to be addressed in a timely fashion. Adapted tele-interventions are reported to have had some success with accompanying benefits. However, to adhere to best practice, the prescription and monitoring of ADHD medication requires regular physical health checks which will need to be completed via face-to-face clinical assessments. The evidence for the efficacy of parent or adult psycho-social tele-interventions is limited. Currently, there is not an evidence base for the effectiveness of remote psycho-social tele-interventions compared with face-to-face interventions for children with ADHD. Due to the nature of this clinical work, it is likely that this will need to be conducted face-to-face. It is also likely that some interventions are more appropriate for tele-health administration than others and post-pandemic interventions may utilise a combination of tele-health and face-to-face interventions.

In addition, many adaptations have been made by educational institutions and young people and their families have experienced a number of difficulties managing remote learning. However, advances in these methods of teaching may provide some future flexibility in learning modalities. Further research is warranted in this area.

### 4.1. Future research

To date, the majority of the research has attempted to identify the difficulties for people living and working with ADHD during this pandemic. However, a few papers have identified possible benefits of this situation. Further research should explore the benefit of remote learning and assessments and interventions conducted via tele-health compared with face-to-face interventions to determine whether they present feasible alternatives for some individuals moving forward. Specifically, research should explore the exact adaptations or mechanisms of intervention that result in beneficial outcomes [66].

There are a number of groups that ADHD research has neglected during this pandemic. For example, adults, older adults, females, black and ethnic minority groups and the LGBTQ+ community. So less is known about how the pandemic has impacted on these groups and what recommendations could be made to better support them. Certain domains are also lacking from the research base, such as how the pandemic has impacted on employment, sexual health, substance use and criminality for those with ADHD.

In addition, thousands of different variants of COVID-19 have been identified throughout the world. Some are reported to have higher transmission, infection and hospital rates [67],[68], such as B.1.617.2, otherwise known as the Delta variant. Although Arbel [65] identified higher recovery rates for people with ADHD in 2020, these findings may not be consistent with the emergence of the Delta variant or other future variants. At the present time, there is little published research into the short-term effects of the Delta variant for young people, adults and older adults and there has not been enough time to determine the long-term consequences of this variant for both ADHD and non-ADHD individuals. Further research should explore the impact of different COVID-19 variants on outcomes for individuals with ADHD.

## 5. Conclusions

The COVID-19 pandemic and worldwide lockdown restrictions have created a number of challenges for all those living and working with ADHD. We are yet to see how service provision will change post-pandemic but it is possible that some adaptations may prove more effective for those with ADHD than previous models of support.
